# Enhanced muscle MRI using deep learning: shorter acquisition time with improved image quality

**DOI:** 10.7717/peerj.21012

**Published:** 2026-03-19

**Authors:** Peilin Fan, Zaizhu Zhang, Bo Hou, Dong Liu, Marcel Dominik Nickel, Jinxia Zhu, Yi Dai, Qian Wang, Fengdan Wang, Wei Yu, Feng Feng

**Affiliations:** 1 Radiology, Peking Union Medical College Hospital, Beijing, China; 2 MR Application Predevelopment, Siemens Healthineers AG, Forchheim, Germany; 3 MR Research Collaboration, Siemens Healthineers Ltd, Beijing, China; 4 Neurology, Peking Union Medical College Hospital, Beijing, China; 5 Rheumatology and Immunology, Peking Union Medical College Hospital, Beijing, China

**Keywords:** Deep learning, Thigh, Magnetic resonance imaging, Diagnostic imaging, Artificial intelligence

## Abstract

**Background:**

This study aimed to evaluate the effectiveness of a k-space learning type deep learning (DL) reconstruction combined with fat-suppressed turbo spin-echo (TSE) T2-weighted imaging (T2WI) in improving image quality and reducing acquisition time for muscle magnetic resonance imaging (MRI).

**Methods:**

In this prospective study, 98 controls (mean age 56.3 years) and 33 patients (mean age 45.2 years) underwent both DL reconstructed TSE (TSE_DL_) and standard fat-suppressed TSE T2WI scans of the bilateral thigh using a 3T MRI scanner. Quantitative metrics, including noise, signal-to-noise ratio (SNR), and contrast-to-noise ratio (CNR), were measured. Two radiologists performed qualitative assessments using a 5-point Likert scale to evaluate image quality, anatomical structure visibility, and diagnostic confidence. Inter-reader agreement and statistical comparisons between groups were analyzed.

**Results:**

The acquisition times were 2 min 11s and 1 min 33s for standard TSE and TSE_DL_, respectively. TSE_DL_ demonstrated significantly lower noise and higher SNR and CNR than standard TSE in both healthy volunteers and patients (*p* < 0.05). Qualitative assessments showed that TSE_DL_ provided superior overall image quality, better visualization of thigh muscles and femoral bones, sharper edges, improved contrast resolution, and higher diagnostic confidence compared to standard TSE (*p* < 0.01).

**Conclusion:**

DL during image acquisition reconstruction, combined with fat-suppressed TSE T2WI, significantly enhances image quality and reduces acquisition time in muscle MRI, suggesting its potential for routine clinical use in musculoskeletal imaging.

## Introduction

Magnetic resonance imaging (MRI) is a multi-parametric and multi-planar technique that provides excellent spatial resolution and soft tissue contrast without the use of ionizing radiation. It plays a critical role in the evaluation of normal and pathological anatomy in the musculoskeletal system (Weaver et al., 2022), particularly for characterizing bone marrow abnormalities, peri-osseous soft-tissue changes, as well as a wide range of muscle and tendon disorders, such as bone marrow edema, tumor infiltration, infection, and inflammatory reactions (Smitaman et al., 2018). Muscle MRI, in particular, is frequently employed for diagnosing conditions such as muscle injuries, edema, and congenital and idiopathic myopathies.

Among the various MRI sequences, turbo spin-echo (TSE) is widely used in clinical workflow due to its ability to offer high-resolution visualization of soft tissues with minimal sensitivity to magnetic field inhomogeneities, especially in high-field MRI (Minssen et al., 2023). However, when applied to large field of view (FOV), such as in bilateral thigh imaging, TSE sequences are often associated with prolonged acquisition times, which can lead to patient discomfort and motion artifacts, ultimately reducing image quality (Marzilger et al., 2019).

In recent years, deep learning (DL) has emerged as a transformative technology in medical imaging. By leveraging large datasets, DL-based reconstruction algorithms can enhance image sharpness and reduce noise during the imaging reconstruction process (Herrmann et al., 2021). DL reconstruction has already shown promising results in reducing scan times while preserving image quality (Recht et al., 2020; Herrmann et al., 2023; Xie et al., 2024; Yilmaz et al., 2023) in brain, spine (Almansour et al., 2023) and knee (Vashistha et al., 2024) imaging. However, its application in large-field musculoskeletal imaging, such as bilateral thigh MRI, remains underexplored. An evaluation of AI products in a prospective cohort with more than 100 patients is particularly rare (Fransen et al., 2025). Moreover, we are just at the beginning of applying AI techniques to reduce imaging time and improve image quality. Caution should be taken since AI algorithms are prone to bias at multiple stages during model development and deployment (Tejani et al., 2024). Their robustness, generalization, and reproducibility needs careful investigation in clinical scenarios.

In this study, we aim to evaluate whether combining a k-space learning type DL reconstruction with fat-suppressed TSE T2-weighted imaging (T2WI) can achieve better image quality and reduce acquisition time in bilateral thigh MRI. By comparing DL-enhanced TSE (TSE_DL_) to standard TSE sequences, we assess both quantitative parameters, such as signal-to-noise ratio (SNR) and contrast-to-noise ratio (CNR), and qualitative measures, including anatomical structure visibility and diagnostic confidence, to determine the clinical utility of this novel approach.

## Materials and Methods

### Study participants

**Figure 1:**
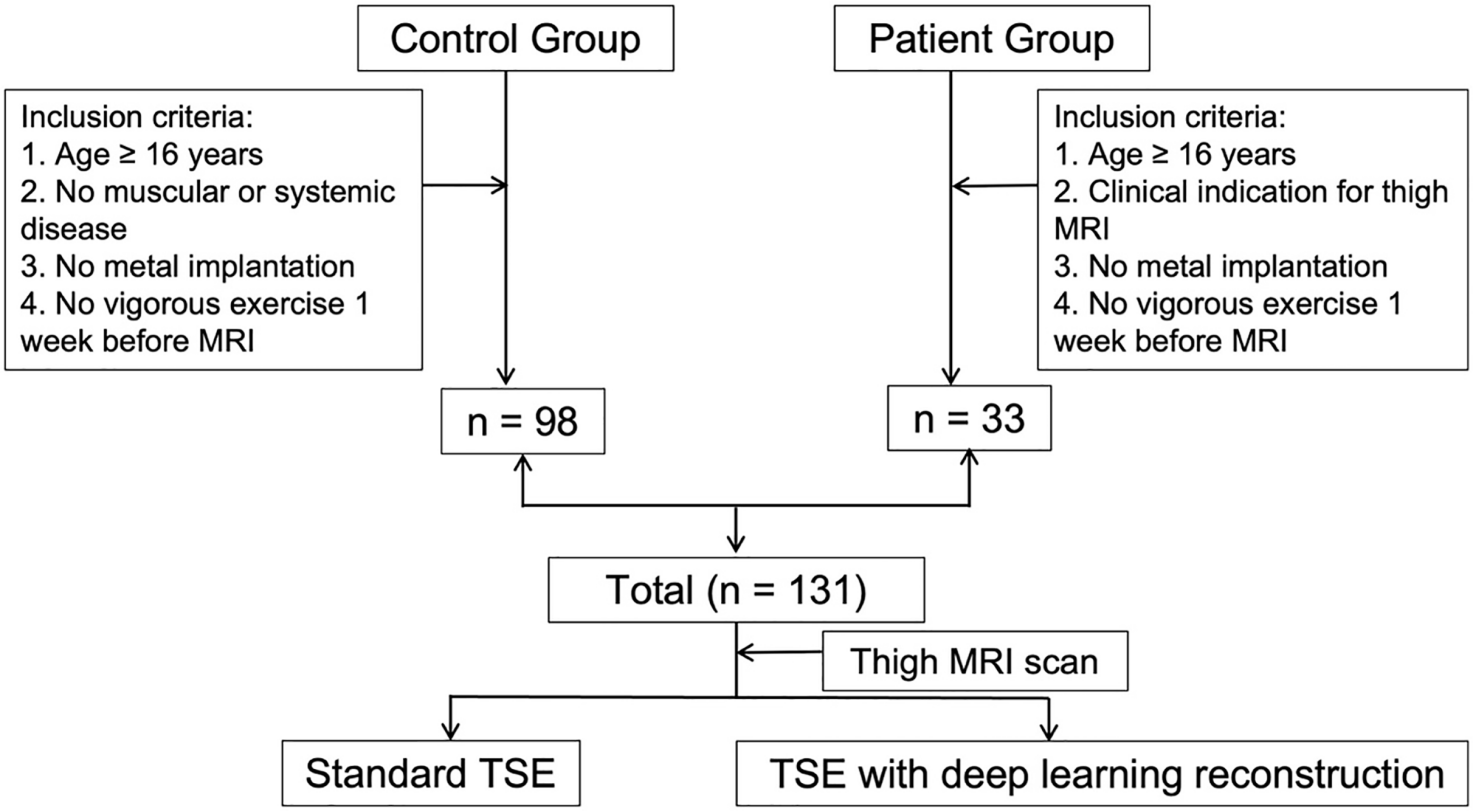
Flowchart shows participants selection. TSE, Turbo spin echo.

This prospective, single-center study was conducted with ethical approval from the Ethics Committee of Peking Union Medical College Hospital, Chinese Academy of Medical Sciences (Approval No. I-23PJ887). Written informed consent was obtained from all participants. Between May 2023 and April 2024, a total of 131 participants were enrolled, comprising 98 healthy volunteers and 33 patients with clinical indications for thigh MRI (Fig. 1).

### Inclusion and exclusion criteria

The participant selection flowchart is presented in Fig. 1. Inclusion criteria for the control group were age ≥16 years; no serious muscular, renal, cardiovascular, neurological, or other systemic disease; no history of surgery or trauma resulting in metal implantation; and no vigorous exercise during the week preceding the study.

Inclusion criteria for the case group were age ≥16 years; no history of surgery or trauma resulting in metal implantation; clinical indication for thigh MRI; and no vigorous exercise during the week preceding the study. Our hospital is a national tertiary medical center, and the final diagnosis was made by rheumatologists or neurologists according to international classification criteria, based on the patients’ clinical presentations, laboratory findings, electromyography, MRI, and biopsy results.

Exclusion criteria for both groups were age <16 years, pregnancy, active breastfeeding, and contraindications to MRI.

### MRI scan protocol

All participants underwent bilateral thigh scans using a 3T MRI scanner (MAGNETOM Vida; Siemens Healthineers AG, Erlangen, Germany) with an 18-channel body coil. Two imaging protocols were applied: a standard TSE fat-suppressed T2WI (standard TSE) and a deep learning-enhanced fat-suppressed T2WI (TSE_DL_). The principle of this DL method combines physical models with data-driven deep learning techniques to enhance the process of MRI image reconstruction. Its core lies in the use of variational networks and hierarchical iterative architecture to reconstruct high-quality images from undersampled data (Herrmann et al., 2021).

Both scans covered the region from the hip joint to the knee joint in axial planes. Key imaging parameters for both sequences included repetition time (TR), 8,180 ms; echo time (TE), 104 ms; FOV, 440 × 303 mm^2^; matrix, 512 × 194; slice thickness, 5 mm; slice spacing, 1 mm; slice number, 50; and phase encoding direction, anterior–posterior. The acceleration factors were 2 and 3 for standard TSE and TSE_DL_, respectively; the corresponding acquisition time for the standard TSE was 2 min 11 s, while the TSE_DL_ scan time was reduced to 1 min 33 s.

### Deep learning reconstruction method

A deep learning–based reconstruction provided by the vendor was used to process acquired, under-sampled multi-coil k-space data. Similar to earlier works (Herrmann et al., 2023; Xie et al., 2024; Almansour et al., 2023; Vashistha et al., 2024), the employed architecture follows a cascaded reconstruction approach, in which convolutional neural network modules are interleaved with data-consistency steps derived from an MRI forward model based on coil-sensitivity encoding. This structure allows the network to enhance image quality while preserving fidelity to the acquired signals, a strategy that has been widely explored for accelerating MRI (Hammernik et al., 2018; Pal & Rathi, 2022; Kalia et al., 2017).

The step sizes for the data consistency updates, along with the parameters of the neural network modules, were optimized through supervised training using pairs generated from fully sampled acquisitions through retrospective under-sampling. For training, a dataset of 25,000 fully sampled slices from healthy volunteers was employed, covering various body regions and a wide range of image contrasts. These data were acquired on 1.5T and 3T systems (MAGNETOM scanners; Siemens Healthineers, Forchheim, Germany). The model was implemented in PyTorch, trained on a dedicated GPU cluster, and subsequently the obtained parameters were exported for integration into the scanner’s prospective reconstruction pipeline.

By design, the algorithm includes a parallel imaging model, allowing to maintain the acceleration factors applied to the standard TSE sequence while reducing residual noise and artifacts. All reconstructions were performed inline, with identical imaging geometry and contrast settings between the standard TSE and the DL-reconstructed TSE sequence.

### Quantitative image analysis

Quantitative image analysis was conducted using the Siemens syngoMMWP VE40A workstation (Siemens Healthineers AG, Erlangen, Germany). Signal intensity (SI) measurements were performed on the adductor muscle in both thighs, with 1-cm^2^ regions of interest (ROIs) placed at three adjacent levels. Noise was measured in air at the same anatomical levels. The SNR was calculated as SNR = SI/noise for each volunteer (Fig. S1). For patients, normal muscle areas on MR images were selected to measure SI and standard deviation (SD) for both sequences; SNR was calculated as described above. Additionally, areas of muscle lesions on MR images were measured to determine SI-lesion; the CNR was calculated as CNR = (SI-lesion – SI)/SD in patients (Fig. S2). Measurements were repeated three times per sequence, and the average values were used for analysis.

### Qualitive image analysis

Two radiologists independently evaluated the image quality using a 5-point Likert scale, focusing on overall image quality, visibility of thigh muscles and femoral bones, edge sharpness, contrast resolution, artifacts, noise, and diagnostic confidence. Radiologist 1 had 15 years of experience in musculoskeletal imaging, and Radiologist 2 had 5 years of general radiology experience. The 5-point Likert scale was 1: very poor anatomical structure visibility, not diagnostic due to very high impacts of artifacts and noise; 2: poor anatomical structure visibility, not diagnostic due to high impact of artifacts and noise; 3: adequate anatomical structure visibility, moderate artifacts and noise, and acceptable image quality; 4: good anatomical structure visibility, low impacts of artifacts and noise, and good image quality; 5: excellent anatomical structure visibility, no impacts of artifacts and noise, and excellent image quality (Kalia et al., 2017).

### Statistical analysis

Statistical analysis was conducted using SPSS Statistics 27 software (IBM Corp, Armonk, NY, USA). Normally distributed data were compared using paired t-tests, while non-normally distributed data were assessed using the Wilcoxon Mann-Whitney test. Noise, SNR and CNR between standard TSE and TSE_DL_ were compared. Inter-rater agreement was assessed using Cohen’s kappa, with kappa values interpreted as follows: 0–0.20, poor; 0.21–0.40, fair; 0.41–0.60, moderate; 0.61–0.80, substantial; 0.81–1, (almost) perfect. The Wilcoxon signed-rank test was used to compare subjective image quality ratings between the two sequences. The threshold for statistical significance was regarded as *p* < 0.05.

## Results

### Participant characteristics

A total of 131 participants were included in the study: 98 healthy volunteers (control group) and 33 patients (case group). The control group comprised 68 women and 30 men (mean age, 56.3 years; range, 45–70 years). The case group consisted of 22 women and 11 men (mean age, 45.2 years; range, 16–69 years). The diagnoses for the case group are summarized in Table 1, which includes conditions such as dermatomyositis, polymyositis, and immune-mediated necrotizing myopathy.

**Table 1:** Patient characteristics.

	Case group
Age	45.2 y (range, 16–69 y)
Female-to-male ratio	2:1
Diagnosis	Dermatomyositis (*n* = 10), polymyositis (*n* = 7), immune-mediated necrotizing myositis (*n* = 2), primary Sjogren’s syndrome (*n* = 1), systemic lupus erythematosus (*n* = 2), ANCA-associated vasculitis (*n* = 1), polymyalgia rheumatica (*n* = 2), Still’s disease (*n* = 1), systemic sclerosis (*n* = 1), myodystrophy (*n* = 4), motor neuron disease (*n* = 1), elevated creatine kinase of unknown origin (*n* = 1)
CK (normal range, 24–70 U/L)	1,009 U/L (range, 13–6,777 U/L)
ALT (normal range, 7–40 U/L)	59 U/L (range, 9–184 U/L)
AST (normal range, 15–35 U/L)	57 U/L (range, 11–145 U/L)
LDH (normal range, 0–250 U/L)	338 U/L (range, 139–668 U/L)

**Note:**

ANCA, antineutrophil cytoplasmic antibody; CK, creatine kinase; ALT, alanine aminotransferase; AST, aspartate aminotransferase; LDH, lactate dehydrogenase.

### Quantitative assessment

**Figure 2:**
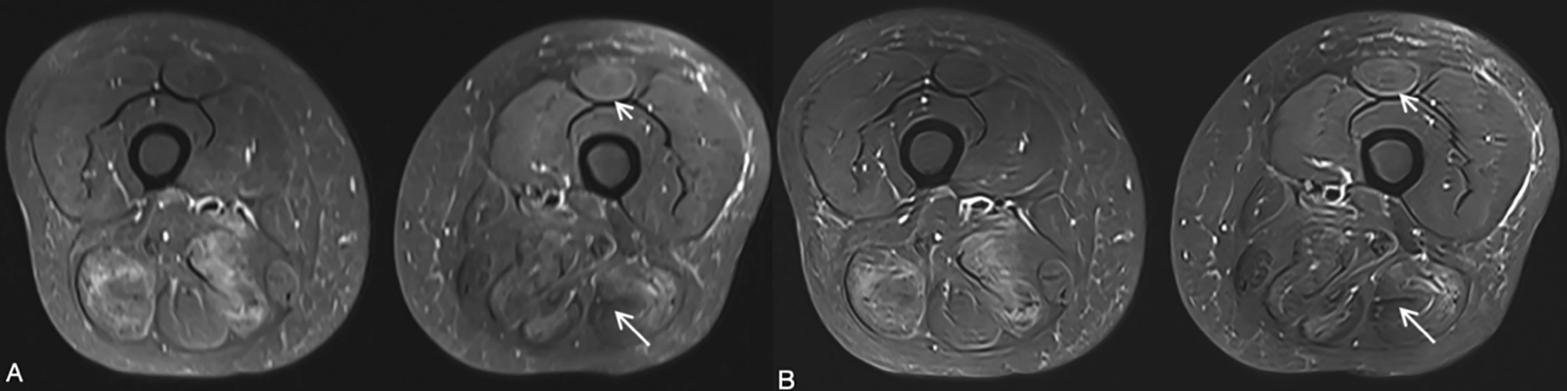
Thigh MRI images of a 48-year-old female patient with immune-mediated necrotizing myopathy. (A) Standard TSE. (B) TSE_DL_. The edge sharpness and contrast resolution of TSE_DL_ image is better than that of Standard TSE image. On TSE_DL_ image, the left biceps femoris tendon (long arrow) is nicely delineated, and fasciitis of the left rectus femoris (short arrow) is evident. By contrast, the left biceps femoris tendon (long arrow) and fasciitis of the left rectus femoris (short arrow) are vaguely shown on standard TSE image.

**Figure 3:**
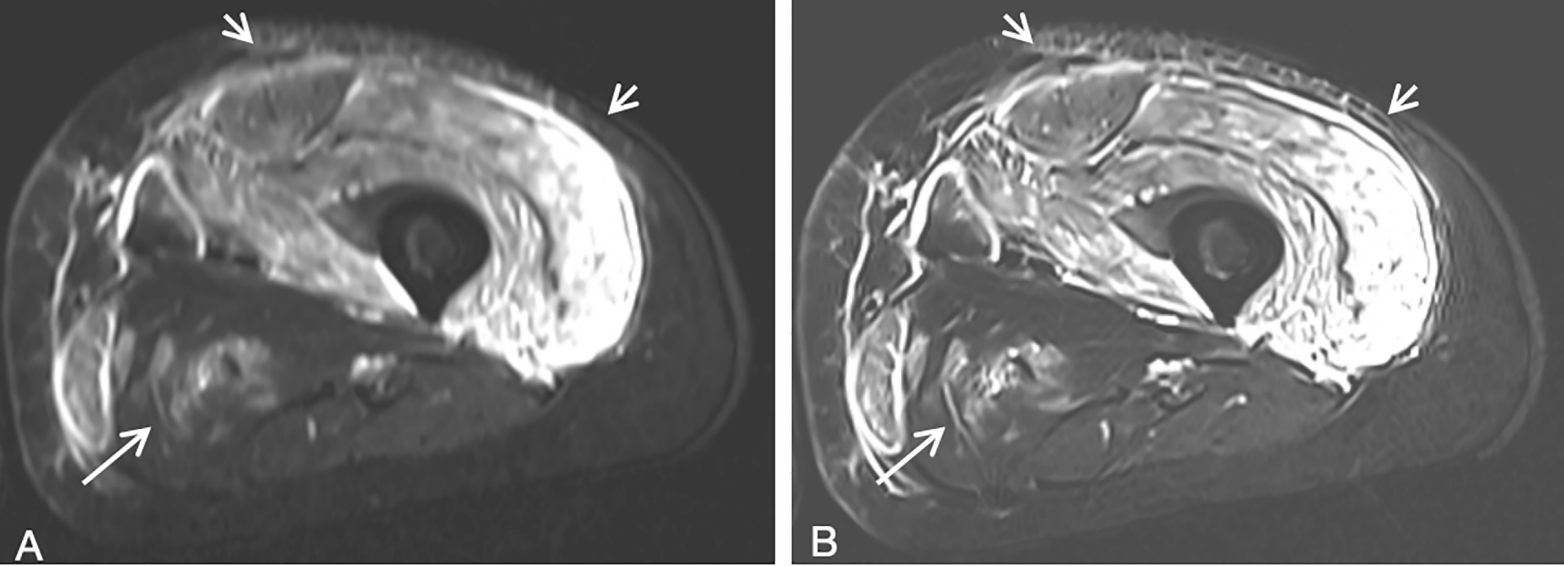
Thigh MRI images of a 30-year-old female patient with dermatomyositis. (A) Standard TSE. (B) TSE_DL_. Marked high signal intensity of thigh muscles on T2WI were observed, especially the quadriceps femoris. Compared with standard TSE image, subcutaneous fasciitis (short arrows), and fasciitis of left thigh muscles including adductor magnus (long arrow) are more clearly shown on TSE_DL_ image, which serves as a key image marker for differentiating dermatomyositis from polymyositis, both of which shared the common features of proximal skeletal muscle weakness and muscle edema.

Quantitative analysis revealed significant improvements in image quality parameters for the TSE_DL_ sequence compared to the standard TSE sequence. Examples of images obtained using both sequences are shown in Figs. 2 and 3. The TSE_DL_ sequence demonstrated significantly lower noise, higher SNR and CNR compared to the standard TSE sequence in both the control and case groups (all *p* < 0.05, Table 2).

**Table 2:** Comparison of quantitative assessment parameters between standard TSE and TSE_DL_ sequence in both control and case groups.

	Group	Standard TSE	TSE_DL_	*p*
Noise	Control	1.14 ± 0.31	0.75 ± 0.11	<0.05
	Case	1.24 ± 0.26	0.92 ± 0.21	<0.05
SNR	Control	60.37 ± 17.96	74.95 ± 18.77	<0.001
	Case	40.43 ± 20.52	50.24 ± 26.40	<0.001
CNR	Case	61.64 (41.20, 95.73)	74.71 (46.65, 123.13)	<0.001

**Note:**

SNR, Signal-to-noise ratio; CNR, contrast-to-noise ratio (non-normally distributed data were presented by IQR, IQR, Interquartile range.).

In control group: The mean SNR for the TSE_DL_ sequence was 74.95 ± 18.77, significantly higher than the SNR of 60.37 ± 17.96 for the standard TSE sequence (*p* < 0.001). In case group: The mean SNR for the TSE_DL_ sequence was 50.24 ± 26.40, significantly higher than the SNR of 40.43 ± 20.52 for the standard TSE sequence (*p* < 0.001). Additionally, the CNR for the TSE_DL_ sequence in the case group was 74.71 (IQR 46.65, 123.13), which was significantly higher than the CNR of 61.64 (IQR 41.20, 95.73) for the standard TSE sequence (*p* < 0.001).

### Qualitative assessment

Cohen’s kappa values indicated substantial to almost perfect agreement between the two radiologists across all qualitative assessment parameters in both the control and case groups (κ = 0.62–0.90 for the control group and κ = 0.63–0.88 for the case group), demonstrating the consistency and reliability of the assessments (Tables 3 and 4). The qualitative assessments showed that the TSE_DL_ sequence consistently outperformed the standard TSE sequence across all measured parameters in both the control and case groups (Table 5).

**Table 3:** Inter-rater agreement between standard TSE and TSE_DL_ sequence in control group.

Parameter assessed	Standard TSE Median score (IQR)		κ	*p*	TSE_DL_ Median score (IQR)		κ	*p*
	Rater 1	Rater 2			Rater 1	Rater 2		
Overall image quality	3 (3–3)	3 (3–3)	0.80	<0.01	4 (4–4)	4 (4–4)	0.84	<0.01
Thigh muscle visibility	3 (3–4)	3 (3–4)	0.84	<0.01	4 (4–4)	4 (4–4)	0.88	<0.01
Femoral bone visibility	4 (4–4)	4 (4–4)	0.83	<0.01	4 (4–5)	4 (4–5)	0.86	<0.01
Edge sharpness	3 (3–4)	3 (3–4)	0.82	<0.01	5 (4–5)	4 (4–5)	0.84	<0.01
Contrast resolution	4 (3–4)	3 (3–4)	0.76	<0.01	5 (4–5)	5 (4–5)	0.75	<0.01
Artifacts	3 (2–3)	3 (3–3)	0.66	<0.01	4 (3–4)	4 (3–4)	0.77	<0.01
Noise	3 (3–3)	3 (3–3)	0.62	<0.01	4 (3–4)	4 (3–4)	0.78	<0.01
Diagnostic confidence	4 (4–4)	4 (4–4)	0.90	<0.01	5 (5–5)	5 (4–5)	0.67	<0.01

**Note:**

IQR, Interquartile range; κ, Cohen’s kappa values.

**Table 4:** Inter-rater agreement between standard TSE and TSE_DL_ sequence in case group.

Parameter assessed	Standard TSE Median score (IQR)		κ	*p*	TSE_DL_ Median score (IQR)		κ	*p*
	Rater 1	Rater 2			Rater 1	Rater 2		
Overall image quality	3 (3–4)	3 (3–4)	0.71	<0.01	4 (4–4)	4 (4–4)	0.73	<0.01
Thigh muscle visibility	4 (3–4)	4 (3–4)	0.81	<0.01	4 (4–5)	4 (4–4)	0.82	<0.01
Femoral bone visibility	4 (4–4)	4 (4–4)	0.63	<0.01	5 (5–5)	5 (4–5)	0.70	<0.01
Edge sharpness	3 (3–4)	3 (3–4)	0.82	<0.01	5 (4–5)	5 (4–5)	0.82	<0.01
Contrast resolution	3 (3–4)	3 (3–4)	0.88	<0.01	5 (4–5)	5 (4–5)	0.75	<0.01
Artifacts	3 (3–4)	3 (3–4)	0.74	<0.01	4 (4–4)	4 (4–4)	0.75	<0.01
Noise	4 (4–4)	4 (4–4)	0.65	<0.01	4 (4–5)	4 (4–4)	0.86	<0.01
Diagnostic confidence	4 (4–5)	4 (4–4)	0.64	<0.01	5 (5–5)	5 (4–5)	0.63	<0.01

**Note:**

IQR, Interquartile range; κ, Cohen’s kappa values.

**Table 5:** Comparison of qualitative assessment parameters between standard TSE and TSE_DL_ sequence in both control and case groups.

Parameter assessed	Standard TSE *vs* TSE_DL_ (control group)		Standard TSE *vs* TSE_DL_ (case group)	
	*p* (Rater 1)	*p* (Rater 2)	*p* (Rater 1)	*p* (Rater 2)
Overall image quality	<0.001	<0.001	<0.001	<0.001
Thigh muscle visibility	<0.001	<0.001	<0.001	<0.001
Femoral bone visibility	0.001	<0.001	<0.001	<0.001
Edge sharpness	<0.001	<0.001	<0.001	<0.001
Contrast resolution	<0.001	<0.001	<0.001	<0.001
Artifacts	<0.001	<0.001	<0.001	<0.001
Noise	<0.001	<0.001	0.008	0.008
Diagnostic confidence	<0.001	<0.001	<0.001	<0.001

In control group: For overall image quality, the TSE_DL_ sequence received a median score of 4 (IQR 4–4), significantly higher than the median score of 3 (IQR 3–3) for the standard TSE sequence (*p* < 0.01). Additionally, the TSE_DL_ sequence provided better visualization of thigh muscles (median 4, IQR 4–4) compared to the standard TSE sequence (median 3, IQR 3–4, *p* < 0.01). The edge sharpness and contrast resolution of the TSE_DL_ sequence were rated significantly higher than those of the standard TSE sequence (*p* < 0.01).

In case group: Similar results were observed in the case group. The overall image quality was rated higher for the TSE_DL_ sequence (median 4, IQR 4–4) compared to the standard TSE sequence (median 3, IQR 3–4, *p* < 0.01). The TSE_DL_ sequence also provided superior visualization of thigh muscles (median 4, IQR 4–5) and femoral bones (median 5, IQR 5–5) compared to the standard TSE sequence (*p* < 0.01). The TSE_DL_ sequence demonstrated significantly better edge sharpness, contrast resolution, and reduced artifacts (*p* < 0.01). As is shown in Fig. 3, Compared with standard TSE image, subcutaneous fasciitis, and fasciitis of left thigh muscles are more clearly shown on TSE_DL_ image, which serves as a key image marker for differentiating dermatomyositis from polymyositis. Furthermore, diagnostic confidence was rated as excellent for the TSE_DL_ sequence (median 5, IQR 5–5) and good for the standard TSE sequence (median 4, IQR 4–4, *p* < 0.01).

## Discussion

In the context of systemic musculoskeletal disorders, large field-of-view MR imaging, such as bilateral thigh MRI, is commonly employed. The spatial resolution and acquisition time are critical factors influencing both image quality and diagnostic confidence. While faster acquisition time could enhance efficiency and patient comfort, they often come at the cost of reduced spatial resolution, which can negatively impact diagnostic accuracy. This study investigated the performance of DL-reconstructed TSE T2WI in thigh MRI in a cohort of >100 patients, demonstrating that TSE_DL_ achieved superior image quality and shorter acquisition times compared to standard TSE sequences.

Previous studies have confirmed the potential of deep learning-based reconstruction to facilitate faster acquisition times while enhancing spatial resolution and maintaining image contrast in musculoskeletal imaging. However, existing research has primarily focused on smaller FOV, such as those in spine and joint imaging, often relying solely on subjective assessments (Herrmann et al., 2021; Almansour et al., 2023; Vashistha et al., 2024). In contrast, our study offers both quantitative and qualitative evaluations, demonstrating that DL-reconstructed TSE T2WI can significantly improve image quality in muscle MR imaging, thereby providing more robust validation for our conclusions. The findings of our study align with earlier research, which showed that DL reconstruction results in lower noise levels when compared to standard TSE sequences (Herrmann et al., 2021; Almansour et al., 2023; Kiryu et al., 2023). Specifically, our results indicated that TSE_DL_ exhibited significantly lower noise scores in the control group. Although the qualitative assessment revealed no statistically significant differences in noise ratings between the two sequences in the case group, the median scores for both raters remained at 4, indicating a generally high quality of imaging. Moreover, the ability of reducing noise by DL reconstruction was more clearly shown by quantitative evaluation (Control group: 0.75 *vs* 1.14, reduced 34%; Case group: 0.92 *vs* 1.24, reduced 26%). This emphasizes the capability of DL algorithms to effectively reduce Gaussian noise (Kiryu et al., 2023; Lundervold & Lundervold, 2019), thereby enhancing the overall image quality and potentially allowing for shorter scanning times.

In the field of MR image reconstruction, deep learning methods can be grouped according to the stage of the imaging workflow in which they are applied. As described in recent publications on DL-based reconstruction (Kiryu et al., 2023), these methods are commonly divided into image-domain methods, k-space–based methods, and direct mapping strategies. Among them, image-domain processing is the most widely used. In this mode, the neural network works after the scanner has completed a conventional reconstruction and is used to improve the final image through procedures such as reducing noise or suppressing artifacts. Since this approach does not depend on the manufacturer’s internal acquisition scheme or reconstruction algorithm, it can be used for images obtained from different MRI systems. Compared with post-processing methods, techniques that involve learning directly in k-space have the advantage of being able to incorporate raw frequency-domain measurements together with information such as phase, coil sensitivities, and noise characteristics. The deep learning method used in our study belongs to this category and served as the early version of what later became the commercial Deep Resolve system. Depending on the reconstruction process, this type of method may begin with signals from individual coils, using sensitivity and noise information as part of the reconstruction, or it may be applied to already combined complex data within the k-space reconstruction procedure.

In addition, k-space learning based DL method is most useful in undersampled parallel imaging applications. Herein, this DL reconstruction was combined with a parallel imaging method called generalized partially parallel acquisitions (GRAPPA) (Deshmane et al., 2012), which utilizes acquired segments of k-space to infer unacquired segments (Hamilton, Franson & Seiberlich, 2017). However, increased acceleration factor of GRAPPA is associated with compromising image quality (Bauer et al., 2011). In this study, an acceleration factor of 2 for the standard TSE sequence and 3 for the TSE_DL_ sequence were used. By adding DL reconstruction, the acquisition time of TSE_DL_ sequence was 29% shorter than that of standard TSE sequence, and with better image quality and diagnostic confidence.

In our qualitative assessments, both readers rated edge sharpness, contrast resolution, and diagnostic confidence significantly higher in the TSE_DL_ imaging group. In previous studies about the comparison between standard TSE and TSE_DL_ image, higher score of edge sharpness in TSE_DL_ imaging was found by Herrmann et al. (2021) and Tajima et al. (2022), but was not by Xie et al. (2024) nor Almansour et al. (2023); no difference was observed regarding contrast resolution (Herrmann et al., 2021) and diagnostic confidence (Almansour et al., 2023). The reasons may lie in that compared with spine and joints, thigh imaging has large volume of muscles in which improved edge sharpness and contrast resolution is more easily observed. Additionally, a more detailed 5-point Likert scale was employed in our study, while all of the other studies used a 4-point Likert scale. The enhanced edge sharpness and contrast resolution provided by TSE_DL_ facilitate better identification of critical anatomical structures and more accurate localization of lesions, ultimately improving radiologists’ diagnostic confidence.

Furthermore, fat-suppressed T2WI MRI is effective in visualizing muscle edema, subcutaneous tissue, and intermuscular fasciitis (Maurer & Walker, 2015; Malartre et al., 2021). Muscle edema is a crucial diagnostic indicator for both congenital and inflammatory myopathies (Pilania & Jankharia, 2022). Fasciitis may represent an early lesion in the progression of dermatomyositis, for histopathological evidence indicates that the fascial microvasculature is a major site of inflammatory cell infiltration in the early stages of dermatomyositis (Spalkit et al., 2021). Our study demonstrated that TSE_DL_ significantly improved visualization of subcutaneous and intermuscular fasciitis compared to standard TSE (Fig. 3). These advancements have the potential to improve diagnostic accuracy in differentiating conditions such as dermatomyositis from polymyositis and congenital myopathy, which share similar clinical presentations.

Despite the promising results, this study had some limitations. First, it was conducted at a single center with a relatively small patient cohort, which may limit the clinical applicability of our findings. Second, the age of participants in the control group was older than that of the case group; however, this is unlikely to affect the results since we evaluate the image quality through self-comparison. Third, this study focused exclusively on axial TSE T2WI; Further studies should include larger, multicenter cohorts and explore the applicability of DL techniques across different imaging planes and sequences. Deep learning reconstruction of under sampled k-space has shown encouraging performance in several musculoskeletal applications, including knee and spine MRI, where scan-time reduction and preserved diagnostic quality have been reported (Recht et al., 2020; Almansour et al., 2023). These findings suggest that similar reconstruction strategies may be applicable to other TSE-based sequences and imaging planes beyond the protocol evaluated in our study. Nevertheless, further multicenter work is needed to confirm robustness across scanners, institutions, and patient populations.

## Conclusions

In conclusion, our findings suggest that deep learning reconstruction of TSE T2WI can provide superior overall image quality and higher diagnostic confidence within a shorter acquisition time for muscle MRI. These results indicate that DL techniques have significant potential for integration into routine clinical practice in the future.

## Supplemental Information

10.7717/peerj.21012/supp-1 Supplemental Information 1 Measurement of SNR for both sequences in control group. (A) Standard T2WI TSE fat-suppressed image. (B) T2WI TSE_DL_ fat-suppressed image. 1 = region of interest (ROI) of normal thigh muscle area; 2 = ROI of air.

10.7717/peerj.21012/supp-2 Supplemental Information 2 Measurement of CNR for both sequences in case group. (A) Standard T2WI TSE fat-suppressed image. (B) T2WI TSE_DL_ fat-suppressed image. 1 = region of interest (ROI) of normal thigh muscle area; 2 = ROI of muscle lesion; 3 = ROI of air.

10.7717/peerj.21012/supp-3 Supplemental Information 3 STROBE checklist.

10.7717/peerj.21012/supp-4 Supplemental Information 4 The original SNR and CNR measurements for the case group.

10.7717/peerj.21012/supp-5 Supplemental Information 5 Reader 1’s Likert scores for image quality assessment in the case group.

10.7717/peerj.21012/supp-6 Supplemental Information 6 Reader 2’s Likert scores for image quality assessment in the case group.

10.7717/peerj.21012/supp-7 Supplemental Information 7 The original SNR and CNR measurements for the control group.

10.7717/peerj.21012/supp-8 Supplemental Information 8 Reader 1’s Likert scores for image quality assessment in the control group.

10.7717/peerj.21012/supp-9 Supplemental Information 9 Reader 2’s Likert scores for image quality assessment in the control group.

## Additional Information and Declarations

### Data Availability

The following information was supplied regarding data availability:

The raw data, qualitative and quantitative measurements for both patients and volunteers, are available in the Supplemental Files.
