# Supplementary material for: Enhanced muscle MRI using deep learning: shorter acquisition time with improved image quality
Source: PeerJ. 2026 Mar 19;14:e21012. doi: 10.7717/peerj.21012 (PMC13006005; doi:10.7717/peerj.21012)
Supplement: Supplemental Information 1 — (A) Standard T2WI TSE fat-suppressed image. (B) T2WI TSEDL fat-suppressed image. 1 = region of interest (ROI) of normal thigh muscle area; 2 = ROI of air. [file peerj-14-21012-s001.pdf]

Appendix

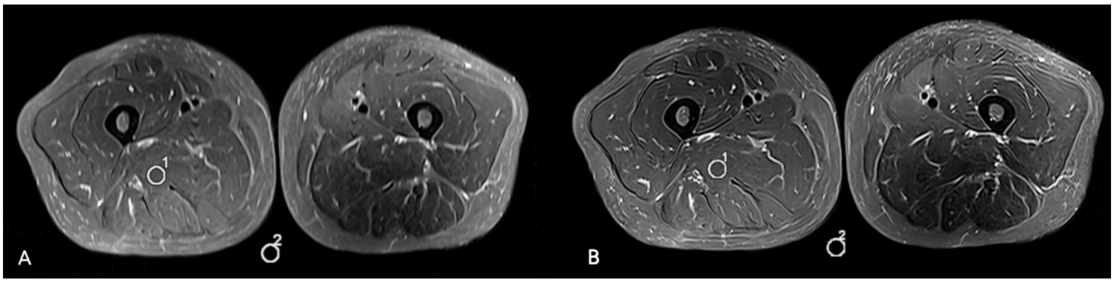

Figure S1 Measurement of SNR for both sequences in control group. (A) Standard T2WI TSE fat-suppressed image. (B) T2WI TSE_{DL} fat-suppressed image. 1 = region of interest (ROI) of normal thigh muscle area; 2 = ROI of air.
